# Cometabolism of ferrihydrite reduction and methyl-dismutating methanogenesis by *Methanosarcina mazei*

**DOI:** 10.1128/aem.02238-24

**Published:** 2025-02-13

**Authors:** Chaojie Guo, Yahai Lu

**Affiliations:** 1 College of Urban and Environmental Science, Peking University 12465 , Beijing, China; University of Illinois Urbana-Champaign, Urbana, Illinois, USA

**Keywords:** ferrihydrite reduction, *Methanosarcina mazei*, ecophysiology, methanogenesis, vivianite, extracellular electron transfer, transcriptome

## Abstract

Recent discoveries have shown that some *Methanosarcina* species can reduce Fe(III), reshaping our understanding of *Methanosarcina* ecophysiology. However, the specific minerals reduced, the products formed, and the underlying metabolic mechanisms remain elusive. Here, we report on the cometabolic process of Fe(III) reduction and methylotrophic methanogenesis in *Methanosarcina mazei* zm-15. Biogeochemical and mineralogical analyses were conducted to investigate Fe(III) reduction from three mineral preparations—ferrihydrite, goethite, and hematite. The results revealed that 38% of the 6 mM Fe(III) in ferrihydrite was reduced within 4 days, and this percentage increased to 75% with the addition of 100 µM anthraquinone-2,6-disulfonate (AQDS). Active Fe(III) reduction occurred immediately and preceded rapid methanogenesis. The addition of ferrihydrite and AQDS together significantly enhanced the maximal CH₄ production rate. However, Fe(III) reduction did not occur in goethite or hematite, even with the addition of 100 µM AQDS. Vivianite was identified as the major product from ferrihydrite reduction. Transcriptomic analysis revealed that gene expression related to the oxidation branch of the methyl-dismutating pathway and the membrane-associated electron transport chain (ETC) was significantly upregulated, whereas the expressions of genes associated with the reduction branch of the methyl-dismutating pathway were downregulated. In conclusion, *M. mazei* zm-15 demonstrates a strong ability to reduce poorly crystalline ferrihydrite, but not highly crystalline goethite and hematite. During the cometabolism of Fe(III) reduction and CH₄ production from methanol, the methyl-oxidation and membrane ETC pathways are enhanced, while the methyl-reduction pathway is downregulated. The mechanism of electron relay from cells to ferrihydrite, however, remains unclear and warrants further investigation.

**IMPORTANCE:**

The recent discovery that certain *Methanosarcina* species can grow by reducing Fe(III) challenges the traditional understanding of methanogens. However, the underlying metabolic mechanisms remain largely unexplored. Using a combination of biogeochemical, mineralogical, and microbiological approaches, we investigated the ability of *Methanosarcina mazei* zm-15. It exhibited a strong capacity to reduce poorly crystalline ferrihydrite but not highly crystalline goethite and hematite. The formation of vivianite from ferrihydrite reduction is likely due to the high rate of Fe(III) reduction and the presence of excess phosphorus in incubations. During the cometabolism of Fe(III) reduction and CH_4_ production from methanol, the methyl-oxidation and membrane electron transport pathways are upregulated, while the methyl-reduction pathway is downregulated. Our research uncovers a differential regulation of metabolic pathways during the cometabolism of Fe(III) reduction and CH_4_ production from methanol. The findings shed new light on the adaptive strategies employed by *M. mazei* in environments with the presence of Fe(III) and suggestthat *Methanosarcina* can play a significant role in methane production and iron cycling in natural environments.

## INTRODUCTION

Methanogens are ubiquitous in the Earth’s anoxic environments and contributes over 60% of global methane emissions into the atmosphere (1, 2). Traditionally, methanogens are separated into two distantly related groups: Class I, comprising *Methanobacteriales*, *Methanococcales*, and *Methanopyrales*, and Class II, comprising *Methanosarcinales*, *Methanomicrobiales*, and *Methanocellales* (3). Class I and Class II methanogens are considered evolved before and after the Earth Great Oxidation Event, respectively (4). Correspondingly, different metabolisms are evolved for methanogenesis, with CO_2_-reducing methanogenesis prevalent across all methanogens, while aceticlastic methanogenesis is present only in the members of *Methanosarcinales* of Class II methanogens. The recent metagenomics-based and cultivation-based studies have revealed a series of novel methanogens outside the traditional Class I and II methanogens (5). For instance, *Methanomassiliicoccales* that are phylogenetically radiated from *Thermoplasmata* are capable of methyl-reducing hydrogenotrophic methanogenesis (6–8). This type of methyl-reducing pathway is also found in *Candidatus* Methanofastidiosa (9), a deep-branching lineage distantly related to Class I methanogens and a few lineages within *Archaeoglobi* previously unknown to methanogenic life (10, 11). Even lineages within the TACK superphylum were found to be capable of methyl-reducing methanogenesis (12–14). Albeit being widespread across different archaeal lineages, the methyl-reducing hydrogenotrophic methanogenesis appears limited to a few specific environments such as hot springs and intestinal systems where methylated compounds are available. In freshwater systems like wetlands, rice paddies, and river sediments, where H_2_, CO_2_, acetate, and other short-chain fatty acids are readily available due to anaerobic decomposition of plant-derived organic matter, methane is produced mainly from H_2_/formate-dependent CO_2_ reduction and aceticlastic methanogenesis (15). The latter process, in particular, accounts for roughly two-thirds of total CH_4_ production (15). Exploring the ecophysiology of *Methanosarcinales* is, therefore, essential for precise evaluation and prediction of CH_4_ emissions from freshwater systems.

Unlike most methanogens that are metabolically monotonous utilizing either CO_2_-reducing or methyl-reducing metabolism, *Methanosarcinales* display a large metabolic diversity. They are capable of utilizing a wide range of substrates including H_2_/CO_2_, formate, acetate, methanol, and methyl-containing compounds (16). A few lineages within *Methanosarcinales* and *Ca*. Methanophagales (i.e., anaerobic methane-oxidizing archaea or ANME) can perform anaerobic CH_4_ oxidation via a mechanism of reverse methanogenesis (17). In terrestrial environments, *Ca*. Methanoperedens can directly use nitrate, nitrite, Fe(III), or Mn(IV) as electron acceptors for methanotrophic growth (18). *Ca*. Alkanophagales and *Ca*. Syntrophoarchaeales, the distant relatives of *Methanosarcinales*, can even anaerobically oxidize multicarbon alkanes (19, 20). The metabolic diversification of *Methanosarcinales* is likely related to their late origin, when diverse habitats and increasing microbial diversity may have facilitated horizontal gene transfer (HGT) (21). A notable evolutionary feature of *Methanosarcinales* is the development of a membrane-associated electron transport chain (ETC) (22). This early form of the ETC includes various components such as F_420_H_2_ dehydrogenase (Fpo), heterodisulfide reductase (HdrDE), cytochromes, methanophenazine (MPH), and MPH-reducing hydrogenase (Vht/Vho). In *Ca*. Methanoperedens, the number of genes coding for multiheme cytochromes (MHC) is as high as in iron-reducing *Geobacter* (23). These ETC components are believed to facilitate extracellular electron transport in *Geobacter* for metal reduction (24) and in ANME for SRB-dependent, nitrate-/nitrite-dependent, and Fe(III)-/Mn(IV)-dependent CH_4_ oxidation (17). Although MHC is occasionally detected in limited numbers, a few typical acetotrophic *Methanosarcina* sp. are also found to perform and benefit from Fe(III) reduction (25–27). Given the importance of these methanogens in freshwater systems, the mechanism of Fe(III) reduction and its environmental implications warrant in-depth evaluation.

Fe(III) reduction by methanogens has been reported for over two decades (28). However, it was only recently proposed that Fe(III) reduction is linked to extracellular electron transport and can support energy conservation (25, 26, 29). The *in vitro* biochemical analysis of *M. acetivorans* indicates that Fe(III) reduction is coupled not only to oxidation of HS-CoB/HS-CoM but also to oxidation of reduced ferredoxin (Fd^2-^) (29). The latter process is catabolized by sodium-translocating complex Rnf, which generates the Na^+^ gradient. Further physiological analysis demonstrates that more energy and larger growth are achieved by reducing ferrihydrite during aceticlastic methanogenesis compared with methanogenesis alone (25). The humic substance can also serve as an electron acceptor. In the presence of the humic substance, methanogenic activity, ATP generation, and cell growth of *M. acetivorans* on either methanol or acetate are significantly promoted, suggesting that reduction of the humic substance occurs similarly to Fe(III) reduction (30). The stimulating effect of the humic substance was also observed in another *Methanosarcina* sp., *M. barkeri* (30). These results indicate that the energy conservation of *Methanosarcina* species is enhanced by respiration on extracellular electron acceptors like Fe(III) and humic substances during methanogenesis. Notably, the test on a humic analog, anthraquinone-2,6-disulfonate (AQDS), revealed that this mode of respiratory growth can occur independently of methanogenesis (31). Recently, it was documented that the methanogenic pathway can work in reverse to support respiratory growth on ferrihydrite or humic substances in both *M. acetivorans* and *Methanococcoides orientis*, a phylogenetically distinct methanogen outside the order of *Methanosarcinales* (26). The reversal of methanogenesis was also reported in *M. barkeri* to support extracellular reduction of ferrihydrite (27). We previously investigated *M. mazei* zm-15, a strain obtained from the Tibetan plateau wetlands (32), and found that the elevated activity of this methanogen was associated with the redox cycling of Fe(III)/Fe(II) in magnetite (33). Furthermore, its growth was significantly promoted by the reduction of iron citrate and ferrihydrite during acetotrophic methanogenesis (34). Altogether, cumulating evidence indicate that *Methanosarcina* species are capable of, and benefit from, iron reduction. However, the mechanism remains elusive. A multiheme cytochrome, MmcA, was found to be associated with ETC and is involved in extracellular electron transport in *M. acetivorans* (25, 31, 35). But the component of this protein varies among *Methanosarcina* species, with seven hemes in *M. acetivorans*, five in *M. mazei*, and its complete absence in *M. barkeri* (36). Furthermore, mutation of this protein does not influence the reduction of humic substances by *M. acetivorans* (30). Clearly, further research is essential to elucidate the mechanism of extracellular electron transport in *Methanosarcina*.

The exceptional consequence of iron reduction by *Methanosarcina* is its impact on iron cycling. A wide range of primary and secondary iron minerals, including ferrihydrite, goethite, and hematite, are prevalent in anoxic soils, natural wetlands, and sediments (37). Seasonal fluctuations of physicochemical conditions, such as water regime, plant growth, and organic input, can induce transformations of iron minerals. For instance, the input of dissolved organic matter (DOM) can favor the formation of water-soluble organic complexes with Fe^3+^, leading to the precipitation of poorly crystalline ferrihydrite, and the alternation of wetting and drying cycles that causes soil redox oscillations can promote the transformation of poorly crystalline ferrihydrite into more highly crystalline minerals such as goethite and hematite (38). Microbial activity can modify the transformation of iron minerals, which has been extensively investigated with *Geobacter* and *Shewanella* (24, 39). However, it remains unknown how Fe(III)-reducing *Methanosarcina* influence iron mineral transformation. Fe(III) (hydr)oxides exist in more than 15 mineral forms, each with different redox potentials and microbial accessibility (37, 40). *G. sulfurreducens* is known to use at least two different inner membrane electron transport pathways to adapt to different redox potentials (41). The reduction of soluble or poorly crystalline ferrihydrite by *Geobacter* occurs easily (42), while the reduction of highly crystallized minerals is more challenging (43). Currently, there is still a lack of research regarding the impact of mineral forms on Fe(III) reduction by *Methanosarcina*.

The purpose of this study was threefold: 1) to analyze the capability of *M. mazei* to reduce Fe(III) in three mineral forms—ferrihydrite, goethite, and hematite; 2) to inform iron mineral transformation by characterizing the products of Fe(III) reduction by *M. mazei*; and 3) to reveal the metabolic insights through global transcriptional analysis of Fe(III)-reducing *M. mazei*. The three iron minerals are widespread in terrestrial anoxic environments and represent different degrees of crystallinity. To evaluate the impact of accessibility to Fe(III), AQDS at a low concentration was applied. A suite of mineral analytical techniques, including X-ray diffraction (XRD), Fourier-transform infrared spectroscopy (FTIR), The Geochemist’s Workbench (GWB), and scanning electron microscopy (SEM), were employed to evaluate the products of Fe(III) reduction.

## RESULTS

### Reduction of Fe(III) during methylotrophic methanogenesis

**Fig 1:**
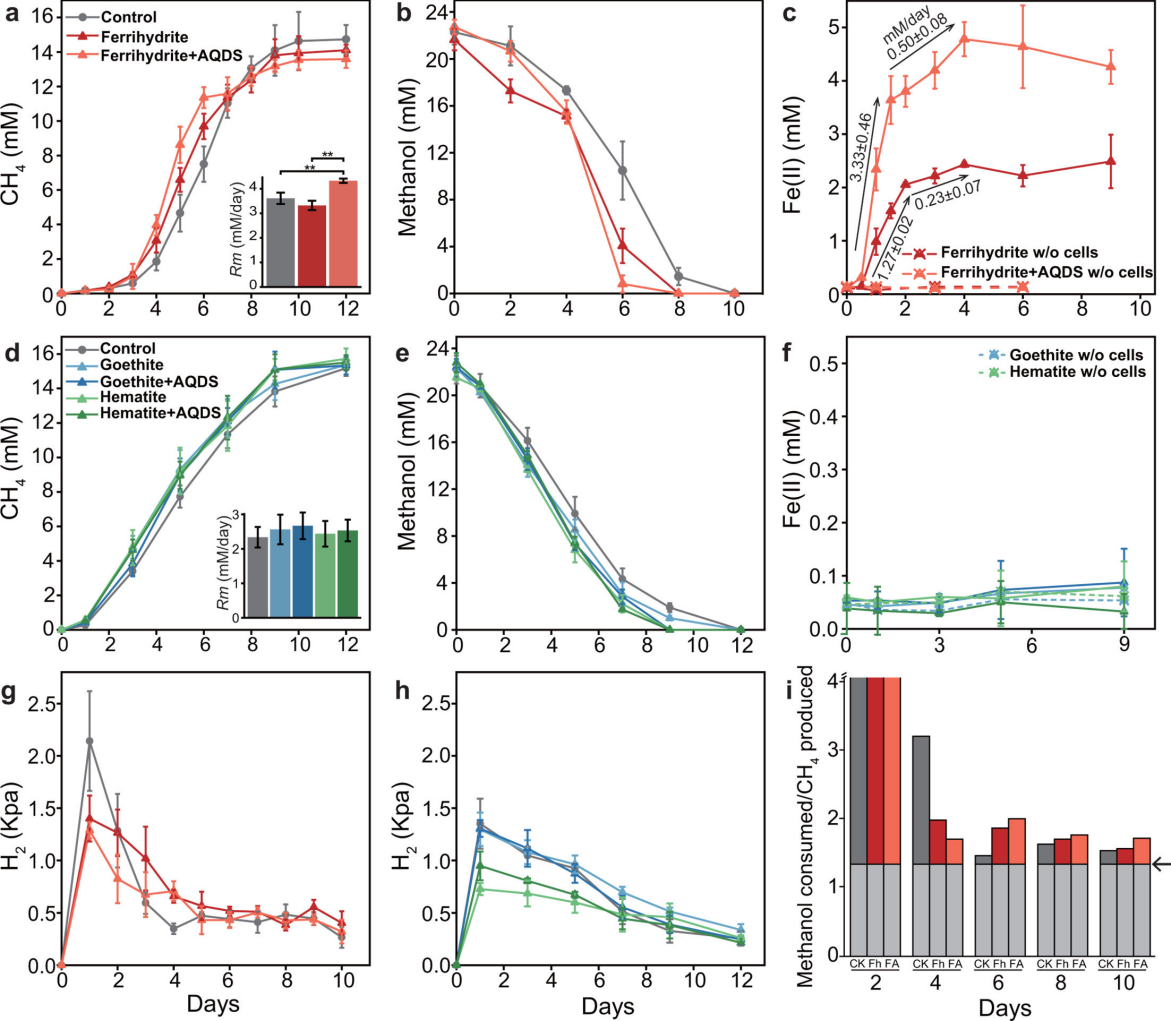
Fe(III) reduction and methanol-dismutating methanogenesis by *Methanosarcina mazei* zm-15. Two experiments were conducted to test Fe(III) reduction from ferrihydrite (Experiment 1: a–c, g,i), and from goethite and hematite (Experiment 2: d–f, h). To facilitate Fe(III) reduction from mineral preparations, 100 µM AQDS was added (denoted as ‘+AQDS’). The figures show the following: (a,d) methane production, (b,e) methanol consumption, (c,f) Fe(II) production, and (g,h) intermediate production of H_2_. (i) For experiment 1, the ratio of methanol consumed to methane produced was estimated, with the black arrow at far right separating the values into stoichiometric fraction (light gray) of methanol-dismutating methanogenesis (ratio of 4:3) and the fraction in excess of stoichiometric ratio (colored based on treatments, dark gray for control, red for ferrihydrite, and orange for ferrihydrite plus AQDS). Note: in (a,d), the CH_4_ concentration was converted from partial pressure in headspace to mmol L^−1^ in the liquid medium using Avogadro’s Law, and the bar-chart insets represent the maximal methane production rates estimated from the Gompertz model; in (c), the black arrows and associated data indicate the Fe(III) reduction rates within the respective periods; in (c,f), the dashed lines represent the abiotic controls without inoculation, i.e. culture medium added with 6 mM Fe in either ferrihydrite, goethite, or hematite, respectively. Error bars represent the standard deviation (SD) of three replicates. Statistical significance was determined using one-way ANOVA, with double asterisks indicating significant differences at *P* < 0.01.

The *M. mazei* strain zm-15 was cultivated in a basal medium with 22 mM methanol as the growth substrate. The cells in the exponential growth phase were inoculated into fresh medium and tested with three iron minerals. CH_4_ production was faster from days 0 to 7 in the presence of ferrihydrite compared with the control culture. The addition of 100 µM AQDS further amplified this effect (Fig. 1a). We fitted the CH_4_ production curve to the Gompertz model and estimated the maximal rate (*R_m_*) of CH_4_ production. This value showed no difference between the ferrihydrite treatment and the control but was significantly greater in the treatment with ferrihydrite plus AQDS, which means the activity of *M. mazei* zm-15 was significantly enhanced by the addition of ferrihydrite and AQDS (Fig. 1a inset). After day 8, cumulative CH_4_ production in the control surpassed that of the iron-supplemented treatments. The consumption of methanol coincided with CH_4_ production (Fig. 1b). The reduction of ferrihydrite Fe(III) occurred almost immediately after inoculation, with the maximal Fe(II) concentration observed on day 4 (Fig. 1c). About 2.2 8 mM Fe(III) was reduced, accounting for 38% of the total ferrihydrite Fe(III) applied, which further increased to 4.5 2 mM (75%) in the presence of 100 µM AQDS. The rate of Fe(III) reduction was 1.27 ± 0.02 mM Fe(II) day^−1^ during the initial 48 hours for the ferrihydrite treatment and 3.33 ± 0.46 mM Fe(II) day^−1^ during the initial 36 hours for the ferrihydrite plus AQDS treatment, respectively. Following this initial phase, the reduction of Fe(III) decreased and leveled off around day 4, whereby CH_4_ production showed a rapid increase. The fresh medium control without zm-15 inoculation showed no reduction of Fe(III), irrespective of AQDS addition (Fig. 1c). Another control composed of zm-15 culture filtrates without living cells also showed no reduction of ferrihydrite Fe(III) (Fig. S1). These results indicate that the strain zm-15 is capable of reducing ferrihydrite, and a small amount of AQDS accelerates this reduction. Notably, the reduction of ferrihydrite Fe(III) occurs ahead of rapid CH_4_ production.

Similar tests were performed with goethite and hematite. CH_4_ production and methanol consumption appeared slightly enhanced by goethite and hematite, but without statistically significant differences (Fig. 1d and e). We also fitted the CH_4_ production curves to the Gompertz model and calculated the maximal rates. The *R_m_* values did not show any statistical difference among treatments, suggesting that the presence of goethite and hematite did not have a significant impact on the growth activity of *M. mazei* (Fig. 1d inset). The analysis of Fe(II) in the culture medium showed almost no reduction of Fe(III) throughout the incubation in all treatments (Fig. 1f). Furthermore, no additional effect was observed with the addition of 100 µM AQDS. The visual appearance of the iron minerals in the culture medium, including their color, remained consistent before and after the incubation. These results indicate that i) the strain zm-15 was not capable of reducing Fe(III) in goethite and hematite, and ii) the methanotrophic methanogenesis was not affected by goethite and hematite.

Hydrogen production was detected in all treatments. The concentration of H_2_ peaked rapidly, reaching maximal values of 1.5–2 kPa on day 1 (Fig. 1g and h). In the later periods, the H_2_ concentration leveled off around 0.5 kPa throughout the incubations. There was no significant difference among all treatments. Given that Fe(III) reduction occurred only with ferrihydrite, we calculated the ratio of methanol consumed to CH_4_ produced in the treatments with ferrihydrite additions. The excess amounts over the stoichiometric ratio of methyl-dismutating methanogenesis (4:3) indicate the consumption of methanol for Fe(III) reduction and cell biosynthesis. The excess consumption of methanol was highest at the beginning and then gradually decreased over the incubation (Fig. 1i). Notably, increased excess consumption of methanol was detected in the treatments with ferrihydrite and ferrihydrite plus AQDS compared with the culture-only control from days 6 to 10. These results indicate that i) a large fraction of methanol is likely used for biosynthesis in the early stages and ii) a part of the excess methanol consumed is used for the reduction of Fe(III) in the ferrihydrite treatments.

**Fig 2:**
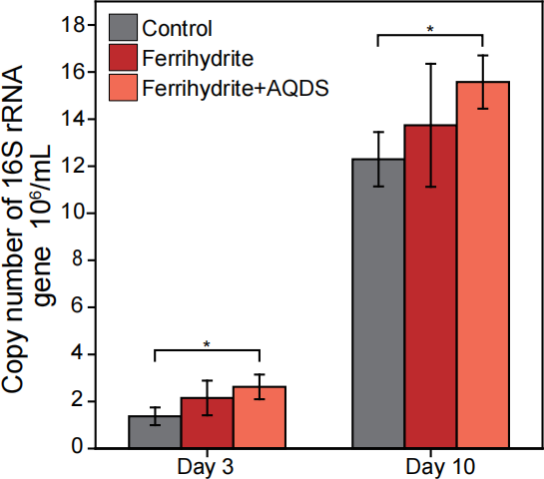
Effect of Fe(III) reduction on growth of *M. mazei* zm-15. The copy number of the 16S rRNA gene in strain zm-15 was quantified using real-time quantitative PCR. This analysis was conducted at two time points during Experiment 1 (see Fig. 1 for detail), where significant Fe(III) reduction occurred from ferrihydrite. Error bars represent the standard deviation (SD) of three replicates. Statistical significance was determined using one-way ANOVA, with single asterisks indicating significant differences at *P* < 0.05.

We measured the growth of strain zm-15 using 16S rRNA qPCR and observed approximately three doublings from days 3 to 10 (Fig. 2). The results showed no difference in cell growth between the control and the ferrihydrite treatment. However, significantly greater growth was observed in the ferrihydrite plus AQDS treatment.

### Characterization of Fe(III) reduction products

Next, we characterized the products of mineral Fe(III) reduction. In the zm-15 culture, the red–brown ferrihydrite particles were transformed into black precipitates (Fig. S2). The addition of AQDS accelerated this blackening, which was followed by gradual decolorization. Abiotic controls without living cells showed no color change over time (Fig. S2).

**Fig 3:**
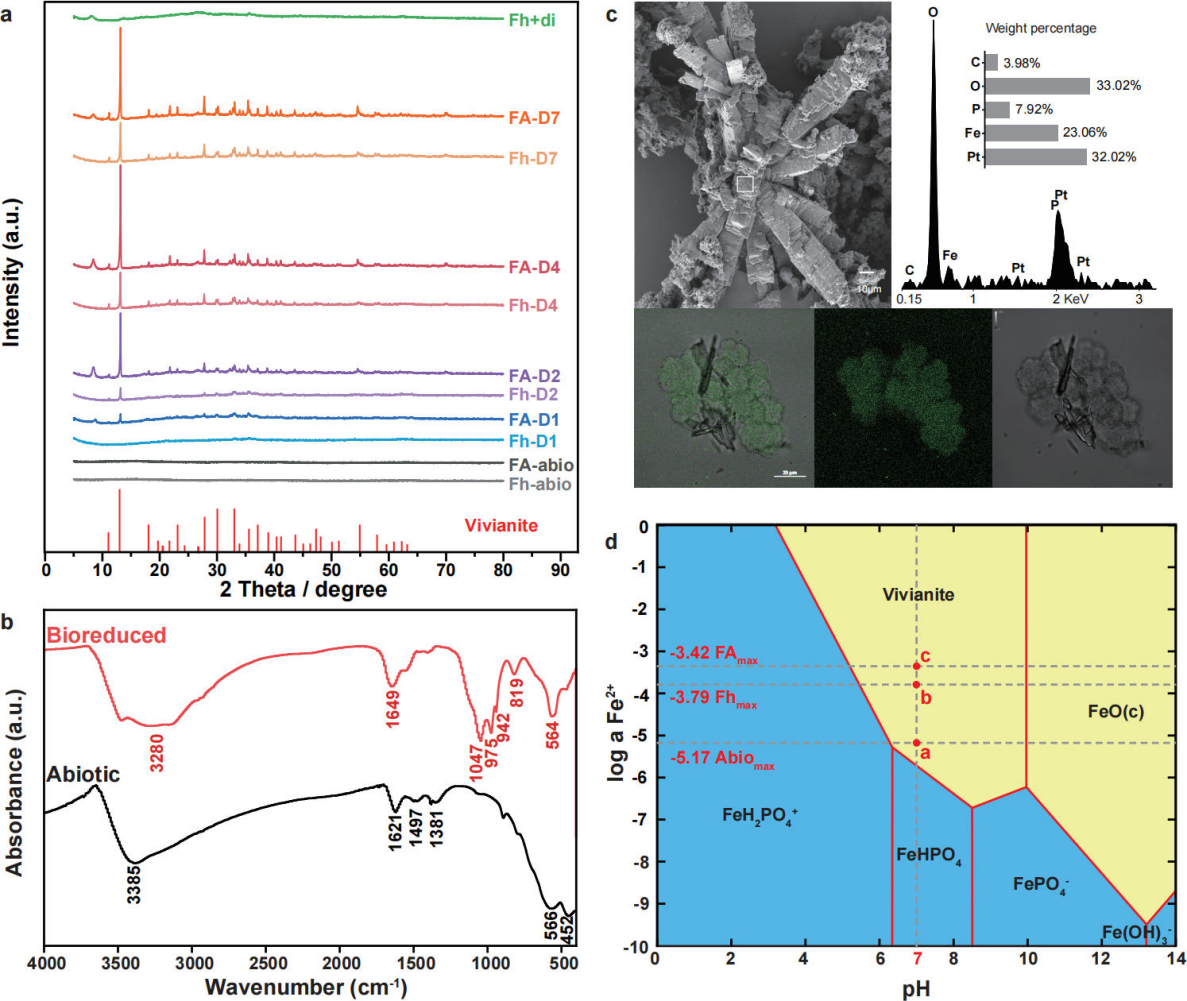
Characterization of Fe(III)-reduced products by *M. mazei* zm-15. (a) XRD spectra of iron mineral particles recovered from zm-15 cultures supplemented with ferrihydrite (Fh) or ferrihydrite plus AQDS (FA). Labels D1, D2, D4, and D7 indicate the time points of sample collection. Three abiotic controls are included for comparison: “Fh-abio” and “FA-abio” represent cell-free incubations with ferrihydrite and ferrihydrite-plus-AQDS, respectively, and “Fh-di” denotes the products after chemical reduction of ferrihydrite with an excess of sodium dithionite. The standard spectrum of vivianite is shown at the bottom. (b) FTIR spectra representing the reduced products from ferrihydrite by strain zm-15 (in red) and an abiotic control (in black). (c) Microscopy observations depicting mineral formation and cell–mineral aggregation. The upper left panel presents SEM images of reduced products of ferrihydrite in the presence of AQDS. The white-boxed region in the SEM image is analyzed via the EDS spectrum (upper right panel), showing significant enrichment of O, Fe, and P, characteristic of vivianite-like formation. The lower panel displays CLSM images, illustrating the formation of cell–mineral aggregates after ferrihydrite reduction, with the autofluorescence of methanogen coenzyme F_420_ shown in green. (d) Solubility diagram of Fe^2+^ versus pH at 30℃ in a simulated system using GWB SpecE8 and Act2 programs under the given reaction conditions of zm-15 cultivations. The three red dots signify critical points of maximum Fe^2+^ concentration under different treatments.

Precipitates collected from cultures at various time points were analyzed using XRD spectroscopy. No characteristic peaks were detected in the abiotic controls (Fig. 3a). However, characteristic peaks emerged gradually over time in the living cultures with ferrihydrite and ferrihydrite plus AQDS treatments (Fig. 3a). The well-developed spectrum from days 4 to 7 matched those of vivianite. The addition of 100 µM AQDS accelerated the appearance of XRD peaks characteristic of vivianite (Fig. 3a), suggesting a faster rate of vivianite formation and higher crystallinity in the presence of AQDS. To confirm that the transformation of ferrihydrite to vivianite is a biological process, we prepared an abiotic control by mixing ferrihydrite solution with an excess of sodium dithionite, a chemical reducing agent commonly used to reduce Fe(III) in the laboratory. Although we observed a color change in the ferrihydrite precipitates, no characteristic peaks were detected (Fig. 3a), indicating the formation of reduced amorphous products. We also conducted XRD analysis for cultures with goethite and hematite at the end of the incubation (Fig. S3). No mineral phase change was evident during methanogenic incubation with goethite or hematite. In the goethite plus AQDS treatment, an additional peak around 13° was detected, possibly indicating partial degradation (Fig. S3). This effect was not observed in the hematite plus AQDS treatment. Chemical reduction by sodium dithionite revealed partial degradation of both goethite and hematite, as indicated by the peaks at 8° and 13°. However, the characteristic spectra of goethite and hematite remained unchanged, indicating no substantial change in mineral phases.

To further characterize products of ferrihydrite reduction by strain zm-15, FTIR analysis was performed over a frequency range of 4,000–400 cm^−1^ (Fig. 3b). The broad absorption bands at 3,385/3,280 cm^−1^ and at 1,621/1,649 cm^−1^ are attributed to the structural OH groups of ferrihydrite. The bands at 1,497 and 1,381 cm^−1^ correspond to Fe–O bonds and Fe–OH bonds, respectively (44). Multiple new bands at 1,046, 975, and 941 cm^−1^ were detected in the reduced products of ferrihydrite by strain zm-15, which are consistent with the P-O stretching vibrations observed in vivianite (45). These results confirm the formation of vivianite as a product of ferrihydrite reduction.

To visualize the mineral morphology and cell–mineral interaction, microscopy observations were performed using SEM-EDS and CLSM (Fig. 3c). SEM images show a predominantly flaky texture of ferrihydrite (Fig. S4a and b). Upon biological reduction of ferrihydrite, numerous degraded pits measuring 1–2 μm in diameter emerged on the mineral surface (Fig. S4c). Methanogen cells were attached to mineral precipitates, forming flocculent microbial–mineral aggregates, with many thin and bladed minerals interspersed within them (Fig. S4d through f). The presence of AQDS increased the crystallinity of the reduced mineral products, resulting in multilayered and radiating prismatic mineral formations (Fig. 3Cc upper-left ; Fig. S4g through i). EDS analysis confirmed the elemental composition of vivianite-like minerals (Fig. 3c upper-right). The aggregation between methanogen cells and newly formed vivianite minerals was also illustrated in CLSM imaging (Fig. 3c bottom). SEM observations for goethite and hematite after methanogenic incubation, however, did not show significant changes in the mineral morphology (Fig. S5).

To enhance our theoretical understanding of mineral transformation, GWB software was used to simulate the precipitation of Fe(II)-minerals under the conditions of present experiments. The simulation diagram suggests that vivianite is the major mineral product across the range of Fe^2+^ concentrations in different treatments (Fig. 3d). The potential maximum Fe^2+^ concentrations produced from Fe(III) reduction are depicted as red dots in Fig. 3d for abiotic reduction control (dot a), culture with ferrihydrite (dot b), and culture with ferrihydrite plus AQDS (dot c), respectively. All potential states are located within the reaction range for vivianite formation.

Altogether, our results indicate that vivianite is the product of ferrihydrite reduction by strain zm-15 under the conditions of present experiments.

### Insights into the metabolic responses

**Fig 4:**
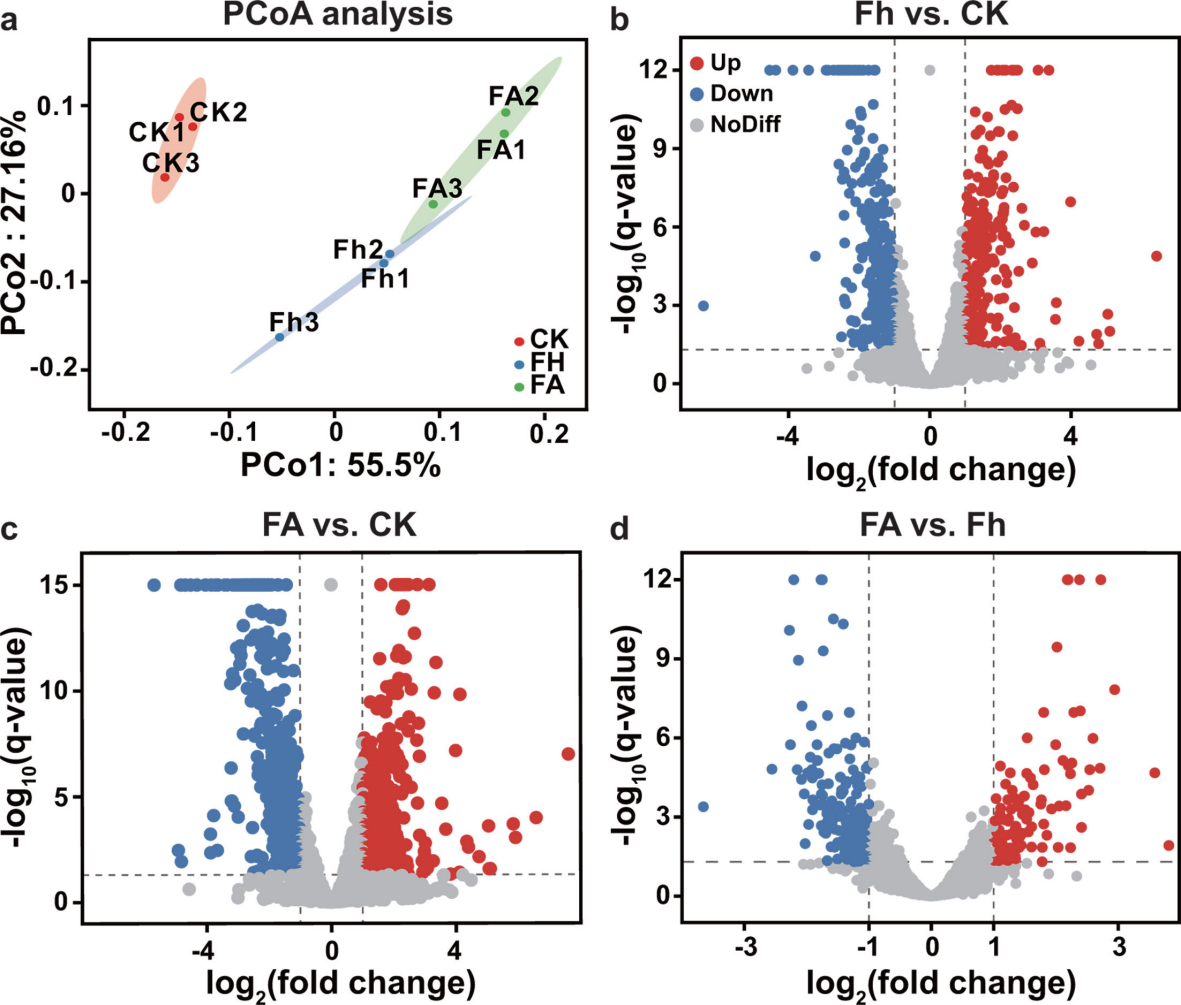
Global transcriptional responses of *M. mazei* zm-15 to ferrihydrite Fe(III) reduction. (a) Principal coordinates analysis (PCoA) of gene expression data, based on the Bray–Curtis distance algorithm, showing clustering patterns for the control (CK), the culture added with ferrihydrite (Fh), and the culture added with ferrihydrite plus AQDS (FA). Each cluster represents three biological replicates. (b,c) Volcano plots illustrating differential gene expression profiles in cultures added with ferrihydrite (b) or ferrihydrite plus AQDS (c) compared to the control culture. The dashed lines represent the thresholds for significant differential expression, defined by |log2(fold change)| ≥1 and FDR-adjusted *P* < 0.05. For visualization purposes, *P*-values lower than 10^−12^ in ferrihydrite culture or 10^−15^ in ferrihydrite-plus-AQDS culture are capped at 10^−12^ and 10^−15^, respectively.

To gain insights into the metabolic responses involved in ferrihydrite reduction, we compared the transcriptomes of cells cultured with ferrihydrite or ferrihydrite plus AQDS against the transcriptome of the control culture without ferrihydrite and AQDS. Principal coordinates analysis (PCoA) on gene expression profiles using the Bray–Curtis distance algorithm reveals the clustering of triplicate samples of the control and each individual treatment (Fig. 4a). However, the expression profiles of the control cultures are separated distantly from those with the addition of ferrihydrite or ferrihydrite plus AQDS, suggesting notable changes in the gene expression due to Fe(III) reduction. The differentially expressed genes are illustrated in volcano plots (Fig. 4b and c). In total, 69 genes and 102 genes were significantly upregulated (log_2_FC ≥ 2, *P* < 0.05), and 55 genes and 125 genes were significantly downregulated (log_2_FC ≤ −2, *P* < 0.05), in cultures with ferrihydrite and ferrihydrite-plus-AQDS, respectively. Additionally, 212 genes and 273 genes were moderately upregulated (log_2_FC = 1–2, *P* < 0.05) and 253 genes and 350 genes were moderately downregulated [log_2_FC = - (1, 2), *P* < 0.05] in the treatments with ferrihydrite and ferrihydrite-plus-AQDS, respectively (Table S2). Based on TPM ranking, the top 20% of highly expressed genes were selected for further analysis. Of these highly expressed genes, 18 and 69 are significantly and moderately upregulated, while nine and 48 genes are significantly and moderately downregulated in the cultures with ferrihydrite, respectively (Table S3). Likewise, 32 and 68 genes are significantly and moderately upregulated, while 19 and 72 genes are significantly and moderately downregulated in the cultures with ferrihydrite-plus-AQDS, respectively (Table S4).

**Fig 5:**
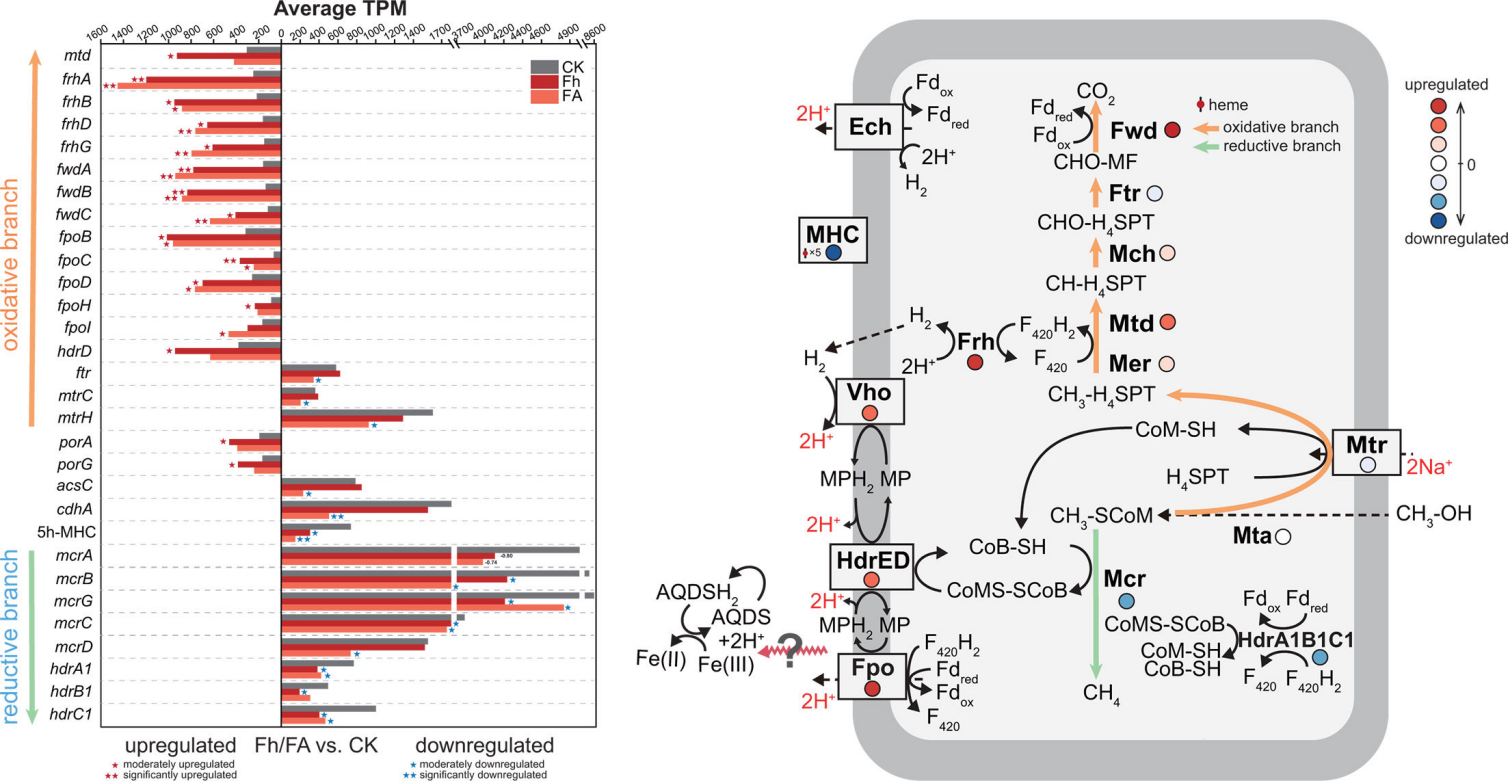
Cometabolic mechanism proposed for Fe(III) reduction and methanol-dismutating methanogenesis by *M. mazei* zm-15. (Left) The bar chart displays the transcripts per million (TPM) values of selected genes that rank within the top 20% of highly expressed genes and exhibit statistically significant differential expressions in the cultures added with ferrihydrite (Fh) or ferrihydrite plus AQDS (FA) compared to the control culture (CK). Single and double asterisks above bars indicate moderate and high levels of differential expression, defined by (|log_2_FC|) within 1 to 2, and ≥2, respectively. Note: for some genes that do not fall within the “moderate” and “high” level categories, the exact log_2_FC values are displayed above the bars instead of using asterisks. For clarity, upregulated genes are positioned on the left and downregulated genes are on the right. The arrows on the far left indicate genes associated with the oxidative branch (in yellow) and the reductive branch (in green) of the methanol-dismutating pathway. (Right) Cometabolic mechanism proposed for Fe(III) reduction and methyl-dismutating methanogenesis. The central metabolic pathway of *M. mazei* is divided into three components: an oxidative branch (yellow arrow), a reductive branch (green arrow), and a membrane-associated electron transport chain (ETC). During the cometabolism of Fe(III) reduction and CH_4_ production from methanol, the oxidative branch and membrane ETC are upregulated, while the reductive branch is downregulated. The color of the closed circles provides a visual summary of the overall gene expression of enzyme complexes involved in the central metabolic pathway. Note that the exact electron relay from the membrane ETC to Fe(III) remains unknown, as indicated by a question mark. Ech, energy-conserving hydrogenase; Frh, F_420_-reducing hydrogenase; Vho, methanophenazine-reducing hydrogenase; HdrED, membrane-bound heterodisulfide reductase; HdrABC, cytoplasmic heterodisulfide reductase; Fpo, F_420_ : methanophenazine oxidoreductase; Mvh, viologen-reducing hydrogenase; Mtr, Na^+^-translocating methyl H_4_MPT-coenzyme-M methyltransferase; 5h-MHC, 5-heme cytochrome c; Mta, methanol-specific corrinoid protein coenzyme M methyltransferase; Mcr, methyl-coenzyme M reductase; Mer, F_420_-dependent methylene-H_4_MPT reductase; Mtd, F_420_-dependent methylene-H_4_SPT dehydrogenase; Mch, methenyl-H_4_MPT cyclohydrolase; Ftr, formylmethanofuran-H_4_MPT formyltransferase; Fwd, formyl-methanofuran dehydrogenase; MF, methanofuran; Fd, ferredoxin; F_420_, coenzyme F_420_; MP, methanophenazine; HSCoM, coenzyme M; HSCoB, coenzyme B; H_4_MPT, tetrahydrosarcinapterin.

Taking the differential expressed genes in the cultures with ferrihydrite and ferrihydrite-plus-AQDS together and focusing on the genes related to methyl-dismutating pathways and biosynthesis, we found that *frhA*, *frhD,* and *frhG* are the most highly expressed and significantly upregulated genes, followed by *fwdA*, *fwdB*, *fwdC,* and *fpoC* (Fig. 5, left). The highly expressed and moderately upregulated genes include *frhB*, *fpoB*, *fpoD*, *fpoH*, *fpoI*, *fpoL*, *hdrD*, *mtd*, *porA,* and *porG*. The *mer* and *mch* are also highly expressed and upregulated, though with low fold changes (Table S3 and S4 ). By contrast, the genes coding for five-heme MHC and the CODH/ACS complex are identified as highly expressed but significantly downregulated (Fig. 5, left). The highly expressed and moderately downregulated genes include *mcrB*, *mcrG*, *mcrC*, *mcrD*, *hdrA1*, *hdrB1*, *hdrC1,* and a series of methyl transfer-related genes, including *mtaA2*, *mtaB2*, *mtaC2*, *mtrC*, *mtrH*, and those coding for methylcobamide-CoM methyltransferase and methylthiol-coenzyme M methyltransferase. Three PQQ-related genes were detected, which, however, showed very less expression and were significantly downregulated (Tables S2). Notably, the genome of strain zm-15 contains four *mtaA* homologs, three of which are highly expressed. Among these, the most highly expressed gene showed no difference among treatments, while the second one was moderately downregulated, and the third one was moderately upregulated (denoted as *mtaA1-4* in Tables S2).

We also compared the gene expression differences between ferrihydrite culture and the culture with ferrihydrite plus AQDS (Fig. 4D). There are 24 and 85 genes that are significantly and moderately upregulated and 11 and 153 genes significantly and moderately downregulated in the presence of AQDS, respectively. Of these highly expressed genes, eleven genes coding for tRNAs and four genes coding for transmembrane transporters are significantly or moderately upregulated, while *mtaB1*, *mtaB2*, *mtaC1*, *cdhA*, *cdhB*, *cdhC*, *cdhD,* and *acsC* are moderately downregulated (Table S6). The upregulation of tRNAs expressions is in line with qPCR analysis (Fig. 2), which showed increased biomass production in the presence of AQDS.

## DISCUSSION

We demonstrated in the present study that *M. mazei* strain zm-15 is capable of reducing ferrihydrite during the methylotrophic methanogenesis. Nearly 38% of 6 mM Fe(III) in ferrihydrite applied was reduced in 4 days, and this percentage increased to 75% in the presence of 100 µM AQDS. The active reduction of Fe(III) occurred ahead of rapid methanogenesis, which leveled off by day 9. However, strain zm-15 was unable to reduce goethite and hematite, even in the presence of 100 µM AQDS. The maximal CH_4_ production rate was promoted by ferrihydrite reduction but was not affected by goethite and hematite. These results suggest that *M. mazei* can reduce poorly crystalline iron minerals but not highly crystallized ones. The function of 100 µM AQDS is likely twofold: serving as an electron transport shuttle and preventing the formation of precipitates on the mineral surface through chemical complexation, thus facilitating ferrihydrite reduction.

The active reduction of ferrihydrite by strain zm-15 is consistent with that in our previous report (34) and aligns with a few reports on other methanogens, including *M. barkeri* and *Methanococcus voltaei* (28), *Methanosaeta thermophila* and *Methanothermobacter thermautotrophicus* (46, 47), and *M. acetivorans* (25). Although the reduction of goethite and hematite by methanogens has also been reported (46, 48, 49), this did not occur under the conditions found in the present experiment. The negative effect of crystallinity on Fe(III) reduction has been well documented in dissimilatory iron-reducing *Geobacter* and S*hewanella* (42, 43, 50). Apparently, the accessibility to Fe(III) is critical to the microbial reduction of iron oxide minerals by bacteria as well as methanogens.

Vivianite is the major product of reduction of ferrihydrite by *M. mazei* in the present experiment. The transformation of ferrihydrite to vivianite by *Geobacter sulfurreducens* has been explored for the development of microbial biotechnology to recover phosphorus from wastewater treatment systems (51, 52). The rate of Fe(III) reduction and the Fe/P ratio are important factors influencing vivianite formation and phosphorus recovery. *G. sulfurreducens* PCA, a model organism in biotechnology applications, exhibited a maximal ferrihydrite Fe(III) reduction rate of 2.29 mM day^−1^ at Fe/P ratio = 1, resulting in the highest vivianite formation efficiency (51). Our experiment revealed a maximal Fe(III) reduction rate of 1.27 mM day^−1^ by *M. mazei*, which increased to 3.33 mM day^−1^ in the presence of AQDS. KH_2_PO_4_ and Na_2_HPO_4_ were provided as pH buffers in our culture medium, with a total P concentration of 12.89 mM. Thus, the relatively high Fe(III) reduction rate and the presence of excess P created a condition favoring the formation of vivianite (Fig. 3). Given the similar Fe(III) reduction rate by and more versatile survival strategies of *M. mazei* in wastewater systems compared to *G. sulfurreducens*, *M. mazei* could provide an alternative for developing wastewater treatment biotechnology. In natural environments, the transformation of ferrihydrite to vivianite may significantly influence Fe and P biogeochemical cycles (53). In agricultural systems such as paddy soils, vivianite formation may help mitigate eutrophication in the downstream aquatic systems by sequestering excess P. On the other hand, vivianite can also serve as a promising slow-release nutrient pool, preventing Fe and P nutrient deficiencies and supporting crop growth (54). However, the P concentration in natural environments may not be as optimal as in the present experiment. Therefore, the *Methanosarcina*-driving iron mineral transformation under natural conditions deserves further investigation.

*M. mazei* utilizes the methyl-dismutating pathway for methylotrophic growth, which consists of an oxidative branch and a reductive branch (Fig. 5, right). The oxidative branch is the reversal of the Wood–Ljungdahl methyl-branch pathway, generating six electrons in the forms of two F_420_H_2_ and one Fd^2-^ per methyl oxidized. These six electrons drive the reduction of three methyl groups into three CH_4_ via the reductive branch. We found that the genes coding for F_420_-reducing hydrogenase (Frh), formylmethanofuran reductase (Fwd), methylene-tetrahydromethanopterin dehydrogenase (Mtd), methylene-tetrahydromethanopterin reductase (Mer), and methenyl-tetrahydromethanopterin cyclohydrolase (Mch) are the most highly expressed and significantly/moderately upregulated under the conditions of ferrihydrite reduction (Fig. 5). The high expression of Frh-encoding genes is corroborated with active H_2_ cycling (Fig. 1g and h). Additionally, genes coding for F_420_H_2_ dehydrogenase (Fpo) and heterodisulfide reductase (HdrDE) were highly expressed and moderately upregulated, and those for methanophenazine-reducing hydrogenase (Vho) were slightly upregulated (Table S2). These results indicate that the expression of most genes for the oxidative branch and part of the membrane ETC is significantly enhanced due to ferrihydrite reduction. Strikingly, the genes coding for the methyl-coenzyme M reductase, indicative of the reductive branch, were highly expressed but significantly/moderately downregulated. The genome of strain zm-15 encodes three soluble heterodisulfide reductases (HdrABCs). Among these, the genes coding for HdrA1B1C1 were highly expressed but were downregulated. In *M. acetivorans*, HdrA1B1C1 is thought to enhance the reduction of CoM-S-S-CoB by utilizing Fd^2-^ without energy conservation, thereby facilitating rapid methylotrophic methanogenesis (55). It is likely that a similar mechanism occurs in *M. mazei*. Altogether, our results indicate that the oxidative branch and part of the membrane ETC are enhanced, while the reductive branch are repressed for ferrihydrite reduction during methylotrophic methanogenesis by *M. mazei*. This conclusion is corroborated by the increased consumption of methanol in ferrihydrite cultures compared to the control culture (Fig. 1i). Notably, the genes coding for pyruvate oxidoreductases (*porA* and *porG*), likely involved in biosynthetic pathways, were upregulated. However, the genes for the CODH/ACS complex, also potentially involved in biosynthesis during methylotrophic growth, were downregulated. Consequently, the regulation of biosynthesis remains unclear, despite the observed increase in growth in the ferrihydrite plus AQDS treatment compared to the control (Fig. 2).

Extracellular electron transfer (EET) is critical for microbial mineral Fe(III) reduction. The EET mechanism has been extensively investigated in the Fe(III)-reducing bacteria, *Geobacter* and *Shewanella* (24, 39). These organisms contain abundant MHCs, which form the nanowire structures responsible for EET (56). Small redox-active molecules such as flavins can also act as electron transfer carriers to relay electrons between microbes and minerals (57). In gram-positive bacteria, extracellular flavoproteins and free flavins together mediate EET (58). A seven-heme MHC, MmcA, is present in *M. acetivorans* (35). Mutation experiments showed that MmcA plays a crucial role in AQDS respiration (31). This MHC is also critical for ferrihydrite reduction during acetotrophic methanogenesis and for energy conservation from Fe(III) reduction (25, 29). A recent biochemical analysis showed that the purified MmcA interacts not only with extracellular Fe^3+^ and AQDS but also with methanophenazine, indicating that MmcA is likely involved in both extracellular and intracellular electron transport (35). Strikingly, *M. acetivorans* mutant strains deprived of MmcA still retained the wild-type’s ability to perform extracellular reduction of humic substances during acetotrophic and methylotrophic methanogenesis (30). Instead of MmcA, the cell surface-associated PQQs were considered to be crucial in mediating the reduction of humic substances (30). The genome of strain zm-15 encodes a five-heme MHC and several PQQs (Table S2). The MHC-encoding gene was highly expressed but downregulated, while PQQ-encoding genes showed very less expression and were downregulated in the cultures with ferrihydrite, indicating that neither MHC nor PQQs are involved in EET. On the other hand, the genes coding for Fpo and HdrDE were highly expressed and upregulated, suggesting that these membrane-bound complexes are likely linked to not only the methanogenic pathway but also EET. However, the details of EET for Fe(III) reduction still remain unknown. Further research employing different approaches, including genetic tools like gene-editing CRISPR technology (59, 60), is essential to elucidate the EET mechanism in *M. mazei*.

### Environmental implications

*Methanosarcina* can utilize a wide range of substrates such as acetate, methylated amines, and methyl sulfides, which are not utilized or less frequently used by other methanogens. Consequently, *Methanosarcina* is one of the most prevalent and dominant methanogens inhabiting various anoxic environments and are significantly responsible for global methane production and emissions into the atmosphere (61–64). Many previous studies have reported that applying iron fertilizers to paddy soil suppresses the activity of methanogens and CH_4_ emissions (65). This effect is due to the increased concentration of Fe(III) in the soil, which acts as an electron acceptor for iron-reducing bacteria. These bacteria are considered to outcompete methanogens for substrates such as acetate and hydrogen, thereby suppressing CH_4_ emissions. From the perspective of redox potential, methanogens typically initiate active CH_4_ production when redox potential (Eh) is below −150 mV (66). The Eh value of goethite and hematite is approximately −290 mV, while that of ferrihydrite ranges from −100 to 100 mV (37). Therefore, iron fertilizers in forms of ferrihydrite may also increase soil Eh, resulting in an inhibitory effect on CH_4_ production.

However, we found that *M. mazei* zm-15 possesses a robust ability to reduce ferrihydrite. *M. mazei* zm-15 was isolated from the Tibetan plateau wetland. The capability of Fe(III) reduction may enable this methanogen to thrive in iron-rich environments and even confer a competitive advantage over iron-reducing bacteria due to its metabolic versatility. Furthermore, strain zm-15 reduces ferrihydrite to vivianite, a mineral with higher crystallinity and a lower redox potential, which may further promote CH_4_ production. While our findings are based on this single strain, thus limiting their broad applicability, similar Fe(IIl) reduction capabilities have been observed in other *Methanosarcina* species like *M. acetivorans* (25), *M. barkeri* (28), and *M. thermophila* (46), implying that Fe(III) reduction might be widespread within the genus *Methanosarcina*. This potential for Fe(III) reduction can expand their ecological niches and metabolic competitiveness across various environments. Further research involving more species and strains is needed to reevaluate the ecophysiology of *Methanosarcina* methanogens in relation to iron and carbon cycling in natural environments. Although the laboratory-based anaerobic incubation in this study does not fully replicate field conditions, our findings also challenge current views on the use of iron fertilizers to mitigate methane emissions from agricultural paddy soils.

## MATERIALS AND METHODS

### Cultivation of *M. mazei* zm-15

*Methanosarcina mazei* zm-15 (CGMCC 1.5193) was kindly provided by Dr. Xiuzhu Dong from the Institute of Microbiology, Chinese Academy of Sciences. This strain was originally isolated from the Zoige wetland located on the Tibetan Plateau (32). It was cultivated in anaerobic serum bottles under 100% N_2_ atmosphere, using DSMZ medium 120, with a slight modification, to exclude original reducing agents in the medium. The basal medium per liter consisted of 0.5 g soybean peptone, 0.5 g tryptone, 0.5 g yeast extract, 0.5 g KH_2_PO_4_, 3.3 g Na_2_HPO_4_·12H_2_O, 0.3 g NaCl, 1.6 g NaHCO_3_, 1 mL mineral solution, and 0.1 mL trace element solution SL-10. The mineral solution contained MgCl_2_·6H_2_O at 2.00 g/L, CaCl_2_·2H_2_O at 0.20 g/L, NH_4_Cl at 6.00 g/L, and NaCl at 6.00 g/L. After autoclaving, the medium was supplemented with vitamins and an Se/W solution in accordance with the medium 120 protocol. Methanol served as the growth substrate, and the cultivations were carried out at 30℃ in the dark.

### Experimental treatments

Experiments were conducted in 50-mL serum vials filled with 20 mL culture medium containing 22 mM methanol. Three iron oxide minerals (ferrihydrite, goethite, or hematite) were prepared to test Fe(III) reduction by strain zm-15. The ferrihydrite and goethite were synthesized *de novo* in the lab, with ferrihydrite synthesized following the method described in (67) and goethite synthesized using the alkaline method detailed in (68). Hematite was purchased from Strem Chemicals (STREM, USA). A humic analog, AQDS (100 µM), was used as an additive to facilitate Fe(III) reduction if necessary. During the experiments, 2 mL of the exponentially growing culture was inoculated into 20 mL fresh medium. The experimental treatments include the following: (i) inoculation with the zm-15 culture only, (ii) inoculation with the addition of 6 mM Fe(III) from each individual mineral preparation, and (iii) inoculation with the addition of 6 mM Fe(III) from each individual mineral preparation plus 100 µM AQDS. Several control incubations were prepared as follows: (i) cultivation of the control culture with the addition of 100 µM AQDS, (ii) fresh culture medium added with 6 mM Fe(III) from each individual mineral preparation, (iii) fresh culture medium added with 6 mM Fe(III) from each individual mineral preparation plus 100 µM AQDS, and (iv) filtrates obtained from the post-growth control cultures added with 6 mM Fe(III) from each individual mineral preparation. All experiments were performed in triplicate.

### Chemical analysis

CH_4_ and H_2_ in the headspace were quantified using a Pressure–Lok precision analytical syringe (Baton Rouge, LA) and analyzed using a gas chromatograph (Agilent 7890B) equipped with a flame ionization detector and a thermal conductivity detector with N_2_ and H_2_ as carrier gases (34). The unit of CH_4_ concentration was converted from partial pressure in headspace to mmol L^−1^ in liquid medium using the Avogadro’s Law (69). For methanol analysis, approximately 100-µL liquid samples were extracted using a syringe, filtered through a 0.22-µm filter, and then analyzed with a gas chromatograph (Agilent 7890 A) equipped with a DB-WAX column (60 m × 0.25 mm [i.d.], film thickness 0.15 µm, J&W Scientific, Folsom, CA) and a flame ionization detector (FID). Helium was used as the carrier gas at a constant flow rate of 1 mL/min.

In this study, we utilized a modified Gompertz equation (equation 1) to assess the potential for methane production (MP), with the assumption that the MP rate is directly proportional to microbial activity within anaerobic systems (70, 71). Specifically, this equation was applied to calculate the kinetic parameters of methanogenesis in the zm-15 cultivations (72):


(1) $$M\left(t\right)=P\times \exp\left[-\exp\left[\frac{R_{m}\times e}{P}\left(\lambda -t\right)+1\right]\right],$$

where *M(t*) denotes the cumulative methane production (mmol L^−1^) at time *t*, *P* refers to the maximum methane production (mmol L^−1^), *R_m_* is the maximum methane production rate (in mmol L^−1^ d^−1^), *e* is approximately 2.718, and *λ* corresponds to the lag phase time (days). The model’s accuracy was quantified using standard errors for kinetic parameters and *R^2^* values from fitting the experimental data. Differences in kinetic parameters among treatments were evaluated for statistical significance using one-way ANOVA at *P* < 0.05.

The concentrations of Fe(II) and total Fe [Fe(tot)] were determined using a ferrozine assay (67, 73). For the Fe(II) measurement, 0.1 mL of culture containing iron oxide particles was stabilized by immediate injection into 0.5 mL of 1 M HCl and incubated in the dark for 24 hours. A 0.1-mL aliquot of acid-stabilized solutions was mixed with 0.9 mL of the ferrozine reagent (1 g L^−1^ ferrozine in 50 mM HEPES buffer, pH 7.0) and measured for Fe(II) concentration using a spectrometer at 562 nm (Microtiter Plate Reader, Eppendorf, Germany). For the Fe(tot) measurement, the sample was mixed with 6 M HCl and shaken at 2,000 rpm overnight at 60℃ to ensure complete dissolution of the iron oxides. The Fe(tot) content was quantified by reducing the sample with hydroxylamine hydrochloride (10% wt/vol in 1 M HCl) for 2 hours in the dark prior to the reaction with the ferrozine reagent.

### Mineral analysis

The zeta potential, particle size, and specific surface area of iron oxide minerals were analyzed according to the method described in (74, 75). The results are presented in the Table S5.

Powder X-ray diffractometer (XRD) analysis was performed to identify the iron mineral species. The culture samples were collected at various time points for the treatments of ferrihydrite and ferrihydrite plus AQDS and at the end of incubations for the goethite and hematite treatments. The samples were centrifuged at 6,000 rpm in 50-mL centrifuge tubes in an anaerobic glovebox. The pellets were collected, air-dried, and ground into fine powders using a mortar and pestle. X-ray diffractometry data were collected on a PANalytical X'Pert PRO X-ray diffractometer using X'Celerator detector equipped with a Cu target (75). The scanning range was set from 5° to 80° 2θ with a step size of 0.02° 2θ and a scanning rate of 1°/min.

Fourier transform infrared (FTIR) spectroscopy was used to characterize the primary functional groups of the ferrihydrite-reduced products. A 20-mL aliquot of the suspension containing the reduced products was collected from the cultivation with ferrihydrite plus AQDS treatment in an anaerobic glovebox. Following centrifugation, the resulting precipitates were air-dried within the glovebox. The dried precipitates were ground into powders using a mortar and pestle. The FTIR analysis was conducted using Nicolet iS5 (Thermo Scientific, USA) with a measurement range covering the mid-infrared region from 4,000 to 400 cm^−1^. An abiotic control was prepared in parallel to collect the FTIR spectra of unreduced ferrihydrite.

To estimate the theoretical products of Fe(III) reduction under the given reaction conditions, simulations were conducted using The Geochemist’s Workbench (GWB, Aqueous Solutions LLC, Urbana-Champaign, USA). The SpecE8 and Act2 programs within GWB were used to calculate the distribution of mass among aqueous species (76).

### Microscopy analysis

Scanning electron microscopy (SEM) was used to observe the morphology of iron mineral species and the aggregation of cells and mineral precipitants. Culture samples were collected by centrifugation, fixed overnight at 4°C in 2.5% (wt/vol) glutaraldehyde, washed with PBS, and eluted with a gradient of ethanol concentrations from 50% to 100% (69). The samples were then coated onto silicon wafers, mounted on SEM stubs using a double-sided tape, and coated with gold using a JFC-1600 sputter coater. The preparations were imaged using a JSM-6700F field emission scanning electron microscope, with the elementary analysis at selected points performed by the equipped INCA energy-dispersive X-ray spectroscopy (EDS) (77). Additionally, a confocal laser scanning microscopy (CLSM, A1R Si laser scanning confocal microscope, Nikon, Japan) was employed to observe the autofluorescence of coenzyme F_420_ in cell–mineral aggregates with the following settings: filter set FITC (fluorescein isothiocyanate), excitation wavelength 467 ± 98 nm, emission wavelength 513 ± 56 nm, and dichromatic mirror wavelength 506 nm (25).

### Real-time quantitative PCR

Culture samples (2 mL) were collected via centrifugation (14,000 g, 20 minutes) and extracted for DNA using the FastDNA Spin Kit for soil (MP Biomedicals, Solon, USA). The copy number of the 16S rRNA gene was quantified using the 7500 fast real-time PCR system (Applied Biosystems) with the primer pair Msm540 (5′-CGGGAAATCTGATAGCTCAAC-3′) and Msm714 (5′-GGTATCTAATCCGGTTCGTG-3′) designed using SnapGene (https://www.snapgene.com). The reaction mixture consisted of 5 µL DNA template, 5 µL SYBR qPCR mix (Toyobo, Japan), 0.5 µL 50 × ROX reference dye, 0.5 µL of each forward and reverse primer, and 6 µL sterilized deionized water. The thermocycling conditions included 95°C for 30 seconds, 40 cycles of 95°C for 10 seconds, 55°C for 30 seconds, and 72°C for 30 seconds, with fluorescence detection at the end of each cycle. The negative control used the sterilized deionized water instead of the DNA template for each qPCR assay. The plasmid DNA standard was prepared with the concentration ranging from 10^3^ to 10^8^ copies of the template per reaction (78). The analysis was carried out in biological triplicate.

### RNA transcriptomic analysis

Strain zm-15 cells at the exponential growth stage from the control culture and the treatments with ferrihydrite and ferrihydrite plus AQDS were harvested from triplicate 25-mL cultures. Cells were split into 50-mL conical tubes (Eppendorf) and pelleted by centrifugation at 10,000 *g* for 15 minutes at 4°C. Pellets were then immediately frozen in liquid nitrogen and stored at −80°C. Total RNA was extracted using TRIzol reagent (Invitrogen) according to the manufacturer’s instructions. DNA digestion, verification of DNA absence, and RNA purification were carried out as previously described (78). The RNA was dissolved in 75 µL RNase-free H_2_O, quality-checked using an Agilent 2100 Bioanalyzer (Agilent Technologies, USA), and quantified using ND-2000 (Thermo Scientific NanoDrop Technologies, USA). High-quality RNA sample (OD260/280 = 1.8 ~ 2.2, OD260/230 ≥ 2.0, RIN ≥ 6.5, 28S:18S ≥ 1.0, >10 µg) is used to construct the sequencing library.

RNA-seq strand-specific libraries were prepared following the instructions of the TruSeq RNA sample preparation Kit from Illumina (San Diego, CA), using 5 µg of total RNA. rRNA was removed using the RiboZero rRNA Removal Kit (Epicenter, USA). mRNA fragmentation, cDNA synthesis, and library preparation were performed using the Illumina TruSeq RNA Sample Prep Kit according to the manufacturer’s instructions. Paired-end sequencing was conducted on an Illumina NovaSeq 6000 sequencer by Shanghai BIOZERON Co., Ltd. Illumina sequence reads have been deposited in the SRA NCBI database with BioProject Accession number PRJNA1175459. Low-quality reads and adapter sequences were removed using Trimmomatic v0.36 (79), and residual rRNA reads were filtered out using BLAST with the Rfam database. Clean reads were aligned to the strain zm-15 genome (accession number in the NCBI database: CP042908) using Rockhopper v2.0.3 with default parameters (80), and uniquely mapped reads were summarized. Differentially expressed genes in the treatments compared to the control culture were identified using DESeq2 with a threshold of |log_2_(fold change)| ≥1 and FDR-adjusted *P* < 0.05 (81). Principal coordinates analysis (PCoA) of gene expression data was performed using “vegan,” “ape,” and “ggplot2” packages in R, based on the Bray–Curtis distance algorithm. Transcripts per million reads (TPM) was calculated to rank gene expression levels within individual samples (82).

## SUPPLEMENTAL MATERIAL

Supplemental figures aem.02238-24-s0001.pdf Figures S1 to S5.

Supplemental tables aem.02238-24-s0002.xlsx Tables S1 to S7.

ASM does not own the copyrights to Supplemental Material that may be linked to, or accessed through, an article. The authors have granted ASM a non-exclusive, world-wide license to publish the Supplemental Material files. Please contact the corresponding author directly for reuse.
