# Supplementary material for: Cometabolism of ferrihydrite reduction and methyl-dismutating methanogenesis by Methanosarcina mazei
Source: Appl Environ Microbiol. 2025 Feb 13;91(3):e02238-24. doi: 10.1128/aem.02238-24 (PMC11921357; doi:10.1128/aem.02238-24)
Supplement: Supplemental figures — Figures S1 to S5. [file aem.02238-24-s0001.pdf]

Supplementary Figures

Cometabolism of ferrihydrite reduction and methyl-dismutating methanogenesis by *Methanosarcina mazei*

Chaojie Guo[a], Yahai Lu[a]#

[a]*College of Urban and Environmental Science, Peking University, Beijing, 100871, China*

#To whom correspondence should be sent:
Yahai Lu, College of Urban and Environmental Science, Peking University,
Yiheyuan Road 5, Beijing, China
Phone: +86 10 62750669
E-mail: luyh@pku.edu.cn

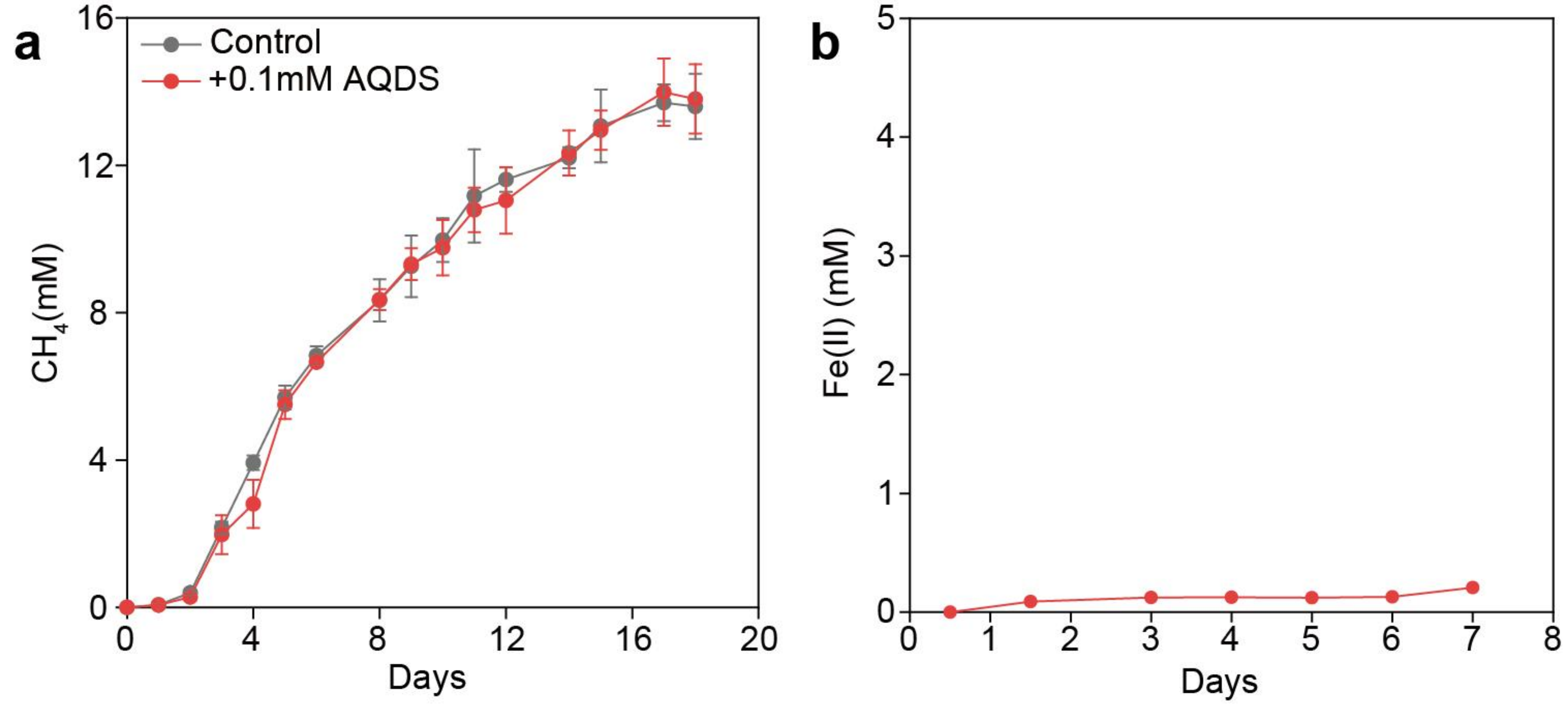

Fig. S1 (a) Methane production over time for methylotrophic *M. mazei* zm-15 with and without 0.1mM AQDS. (b) Fe(II) concentration over time in the filtrate of zm-15-cultured medium with the addition of 6 mM ferrihydrite.

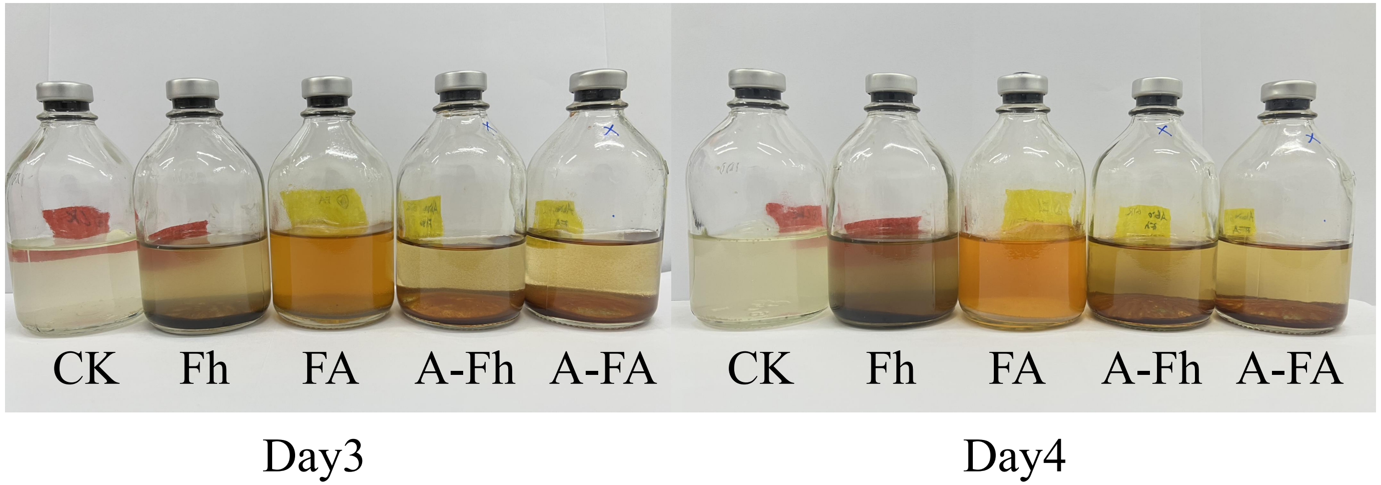

Fig. S2 Change in color of the cultures. CK, cells with no additon; Fh, cells with 6mM ferrihydrite; FA, cells with 0.1mM AQDS and 6mM ferrihydrite; A-Fh, Abiotic control with 6mM ferrihydrite; A-Fh, Abiotic control with 0.1mM AQDS and 6mM ferrihydrite.

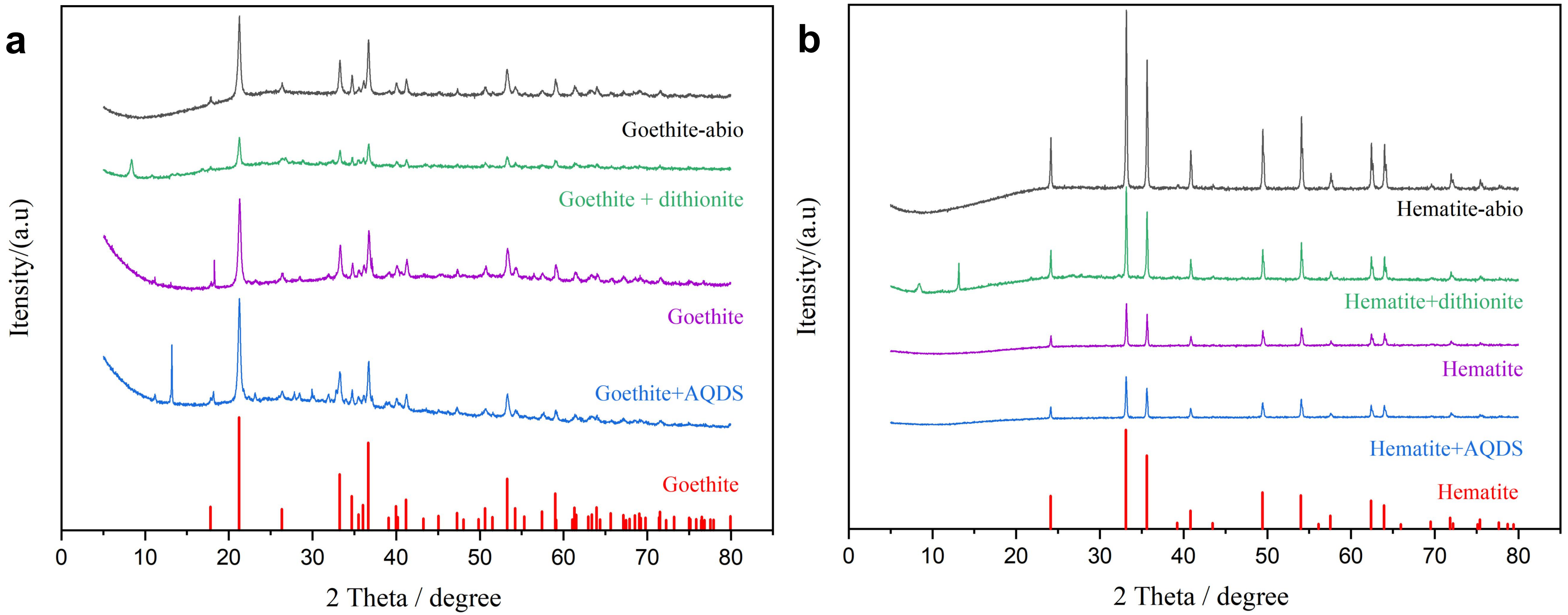

Fig. S3 XRD spectra of iron oxide particles recovered from cultures at various time points in addition of goethite (a) and hematite (b).

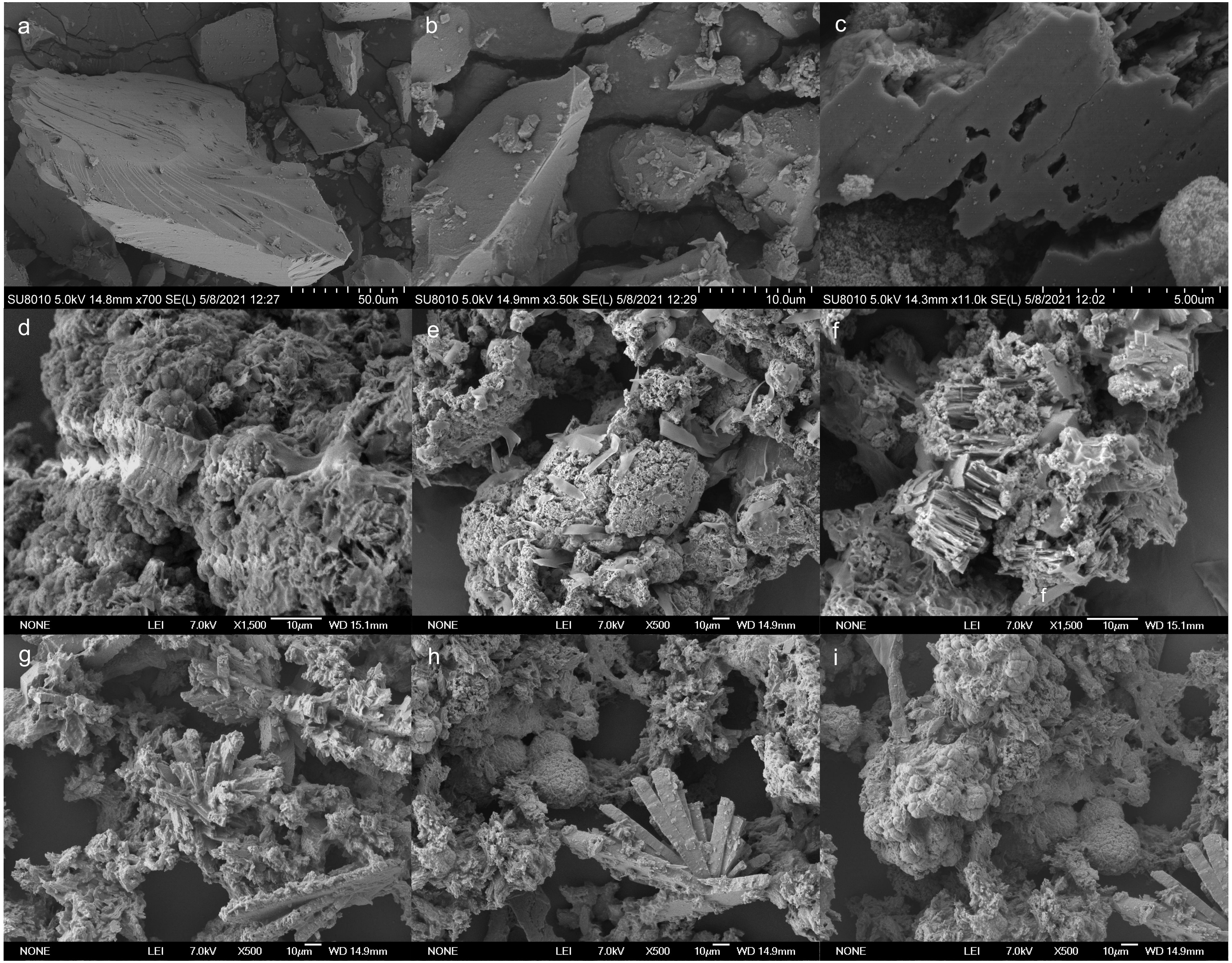

Fig. S4 SEM images of abiotic control of ferrihydrite (a, b), dissolution pits in bioreduced ferrihydrite of microbial size (c), bioreduced ferrihydrite (d, e, f), bioreduced ferrihydrite in the presence of AQDS (g, h, i) by *M.mazei*.

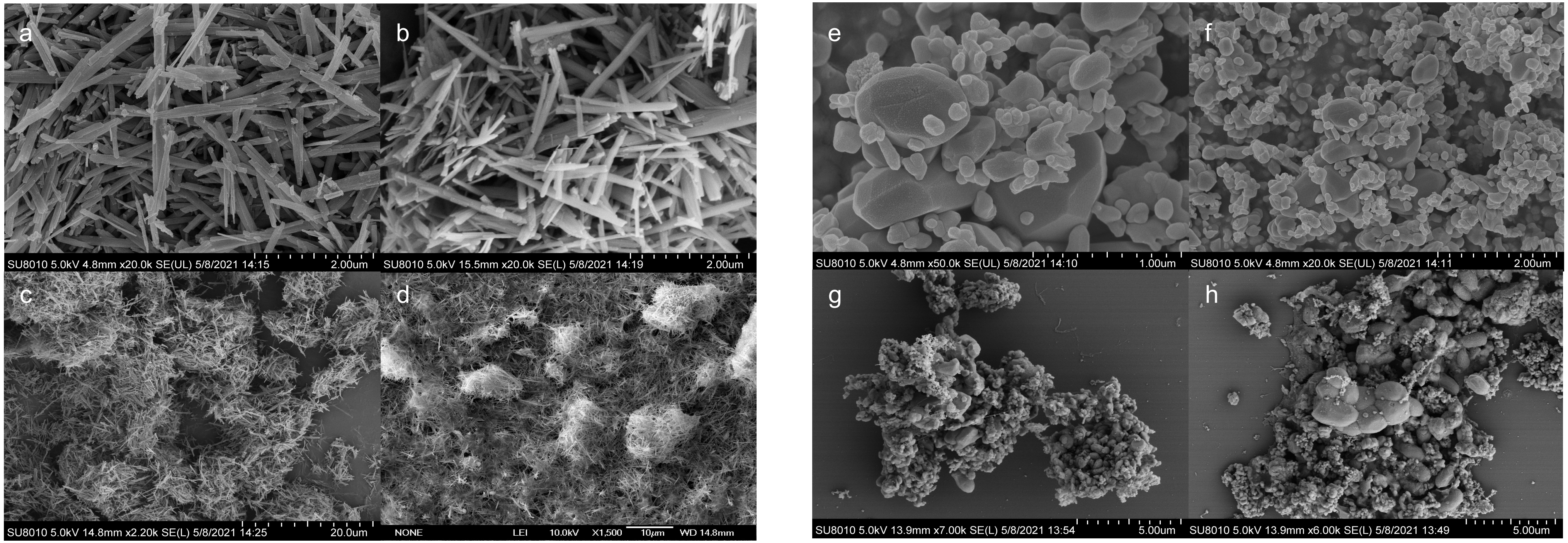

Fig. S5 SEM images. The left side of the figures (a, b, c, d) illustrates the treatment related to goethite, while the right side (e, f, g, h)corresponds to the treatment associated with hematite: (a, b and e, f) goethite and hematite without biological treatment; (c, d and g, h) goethite and hematite after microbial cultivation. No apparent mineralogical phase transformation was observed.
